# Beta-propeller protein-associated neurodegeneration: A clinical update with a case report

**DOI:** 10.1016/j.ensci.2023.100469

**Published:** 2023-06-08

**Authors:** Moustafa A. Mansour, Yehia Moawad, Hassan Ali

**Affiliations:** a Department of Neurology and Neurologic Surgery, Faculty of Medicine, Al-Azhar University, Cairo, Egypt; b Department of Neurology and Neurologic Surgery, Mayo Clinic, Rochester, MN, USA; c Division of Neuro-Intensive Care, Dar Al-Fouad Medical Corporation, Cairo, Egypt; d Department of Emergency Medicine and Critical Care, Faculty of Medicine, Al-Azhar University, Cairo, Egypt; e Department of Pediatrics, Faculty of Medicine, Al-Azhar University, Cairo, Egypt; f Division of Neurology and Neurodevelopmental Disorders, Department of Pediatrics, Faculty of Medicine, Al-Azhar University, Cairo, Egypt

## Abstract

• BPAN is a phenotypically distinct, X-linked form of NBIA caused by an error in autophagy due to a WDR45 gene mutation.
• Patients with BPAN present with a global developmental delay that remains relatively static until adolescence/young adulthood.
• A unique frameshift mutation in the WDR45 gene has been identified in our case, a mutation previously only reported in a single BPAN case.
• Unlike the first case published in 2013, our patient had no dysmorphic Rett-like features, and her mutation was confirmed as a *de novo* mutation.
• Some previous studies reported a correlation between the detected WDR45 mutations and responsiveness to L-Dopa, but with no available data regarding the variant NM_007075.3: c.186delT, p.L63Wfs*19, making our study the first to report such a finding.

## Graphical abstract

**Figure:**
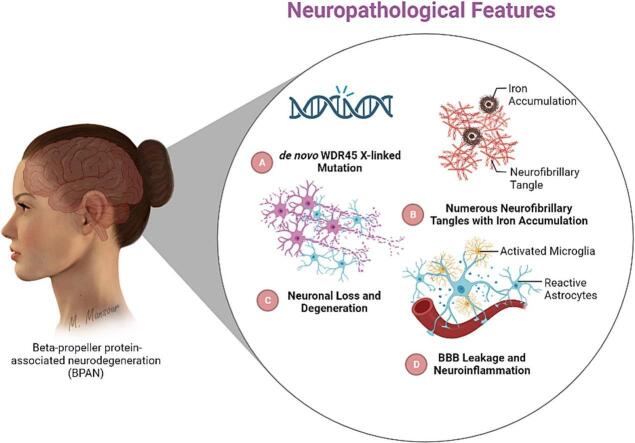

Dear Editor:

Recently defined, BPAN is the only X-linked subtype of neurodegeneration with brain iron accumulation (NBIA) due to a *de novo* mutation in the WD repeat domain 45 (WDR45) gene at the Xp11.23 locus, which is responsible for encoding a beta-propeller scaffold protein, hence the name BPAN [1,2]. In BPAN, patients present with global developmental delay, including delayed language and motor skills, and unlike other subtypes of NBIA that can be manifested early, the clinical presentation of delayed development remains relatively static until adolescence/young adulthood in BPAN [3]. Cases that typically describe BPAN are rarely reported due to the extremely low prevalence of the major NBIA entity (∼1/1,000,000) with BPAN only constituting approximately 5–7% of these cases, in addition to the considerable overlap with other subtypes of the NBIA entity [4].

This is a 29-year-old woman who presented to the neurology clinic with a two-year history of gradually worsening bradykinesia, tremors, rigidity, dystonia, dysphagia, and severe neurocognitive decline. She had no family history of any neurological disorders. Her family stated that she had a severe global developmental delay in early childhood, along with intellectual disability. Although her course of intellectual disability was static for an extended period, the family noted a history of recent neurocognitive decline. A general examination showed an obese young female with short stature. On neurological examination, severe neurocognitive decline and poor language skills were evident (her communication was mainly through inarticulate sounds and gestures). A marked rigidity was assessed in all extremities, and a pronounced posture (dystonic posturing with tremors) was noted. Bradykinesia and dysphagia were also evident, and meticulous examination did not reveal any dysmorphic features. Magnetic resonance (MR) imaging was performed (Fig. 1) for further follow-up. Axial T2-weighted MR image at level of basal ganglia demonstrated mild diffuse brain atrophy as suggested by prominent ventricles and sulci. A hypointensity involving bilateral globi pallidi was also noted (**A**, *arrows*). Axial T2-weighted MR image at level of substantia nigra demonstrated hypointensity involving substantia nigra and subthalamic nuclei on both sides (**B**, *arrows*). Axial T1-weighted MR image at level of substantia nigra demonstrated bilateral symmetric, high signal involving the substantiae nigrae, with a centrally intervening band of hypointensity (**C**, *arrows*) virtually pathognomonic of BPAN [2]. Susceptibility-weighted imaging (SWI) could not be performed. Whole-exome sequencing (WES) was performed, which identified a heterozygous frameshift mutation in the WDR45 gene: c.186delT, p.L63Wfs*19. To our knowledge, this mutation has only been reported in a single case with a confirmed BPAN diagnosis [2], making our case the second one. In contrast to the first case that was published in 2013 [2], our patient did not exhibit any dysmorphic Rett-like features and we confirmed that her mutation to be a *de novo* mutation by the negative testing in the biological parents. Confirming a *de novo* mutation in the first reported case could not be achieved (reported as unknown) [2].

**Fig. 1:**
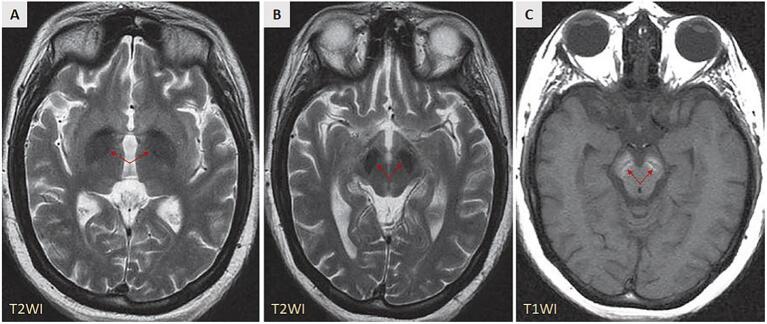
[**A**] Axial T2-weighted MR image at level of basal ganglia demonstrates hypointensity involving bilateral globi pallidi (*arrows*). [**B**] Axial T2-weighted MR image at level of substantia nigra demonstrates hypointensity involving substantia nigra and subthalamic nuclei on both sides (*arrows*). [**C**] Axial T1-weighted MR image at level of substantia nigra demonstrates bilateral symmetric, high signal involving the substantiae nigrae, with a central band of hypointensity (*arrows*).

After the diagnosis of BPAN had been confirmed in our patient, the patient was started on combined levodopa/carbidopa (100/25 mg, *QID*) which led to substantial improvement in motor functions (particularly, improvement of the bradykinesia), language skills (slight vocabulary improvement), and her general condition.

## 1 Brief discussion

Global developmental delay, early childhood neurocognitive decline that remains static for years, juvenile or young-adult onset of worsening neurocognitive changes, and the development of progressive, levodopa-resistant Parkinsonian features suggest the diagnosis of beta-propeller protein-associated neurodegeneration (BPAN), which is the only X-linked subtype of neurodegeneration with brain iron accumulation (NBIA) [[1], [2], [3], [4]]. In the past, this entity was named static encephalopathy (of childhood) with neurodegeneration in adulthood (SENDA), but after the underlying genetic defect was identified, this NBIA subtype was renamed or reclassified as BPAN, similar to the other subtypes of NBIA [5]. In BPAN, and as discussed earlier, patients present with global developmental delay, mainly including language and motor skills. In contrast to other subtypes of NBIA, the clinical presentation of delayed development remains relatively static until adolescence/young adulthood [3]. Abnormal movement or neurocognitive decline is absent in childhood, but as the patient approaches adolescence and early adulthood, progressive neurodegeneration symptoms including dystonia, Parkinsonian features, and neurocognitive decline start to appear [2,3]. Although the benefit is only short-lasting, Parkinsonian symptoms may improve with the initiation of levodopa therapy as our patient did [6]. Some previous studies reported a correlation between the detected WDR45 mutations and responsiveness to L-Dopa [2,7], but with no available or unknown data regarding the variant NM_007075.3: c.186delT, p.L63Wfs*19, making our study the first to report such a finding (i.e., positive response to L-Dopa).

As in other subtypes of NBIA, iron deposition is the key imaging abnormality [8]. The earliest and most concentrated iron accumulation occurs in the substantia nigra. Although iron deposition generally occurs in the globus pallidus, it usually follows iron deposition in the substantia nigra. Therefore, evidence of increased iron deposition involving bilateral substantiae nigrae as demonstrated by the high T1 signal and the low T2 signal is virtually a pathognomonic finding in patients with BPAN [8].

Late-onset pantothenate kinase-associated neurodegeneration (PKAN) may be considered in the differential diagnosis of BPAN; however, the typical eye-of-the-tiger sign in the globus pallidus, which is reported as a specific sign of PKAN [9], is absent in our case. Moreover, the T1 hyperintensity of the subthalamic nucleus (STN), as seen in our case, is not characteristic of PKAN [8,9]. Phospholipase-associated neurodegeneration (PLAN), which may also be considered in the differentials, is a disease of early childhood and is characterized by more severe symptoms, most notably cerebellar atrophy which is not present in our case [10]. Furthermore, most patients with PLAN succumb to the disease by 10 years of age [10]. While our patient's symptoms complex is also suggestive of Parkinson's disease, especially with the marked response to L-Dopa, the evidence of excessive iron accumulation in the deep nuclei is not a feature of early-onset Parkinson's disease [11].

BPAN is thought to be caused by an error in autophagy, the process of cellular recycling [12]. Thus far, several research groups have been working to unveil how a disruption in this process leads to iron build-up in the brain, and consequently the characteristic clinical phenotype. To date, no targeted therapies are approved or readily available for this condition, with most current treatments directed toward the manifested symptoms; however, we and the families of our BPAN patients remain hopeful that new targeted therapies may become available shortly as we learn more about this rare disease.

## Ethics approval and consent to participate

The approval was obtained from the Ethics Committee of Al-Azhar University Hospitals [REF. HSUZ-21-00032711]. The patient's guardians gave a written informed consent to publish the case and any related data.

## Funding

None declared.

## Contribution

M.M. was responsible for the conception of the work, data collection, drafting the article, critical revisions, illustrating the abstract, and obtaining approval of the final version of the manuscript. Y.M. contributed by drafting the article, and critical revisions. H.A. contributed by critical revisions of the article. All authors read the final manuscript and were involved in direct patient care.

## Declaration of Competing Interest

None declared.
